# Gas Signaling Molecules and Mitochondrial Potassium Channels

**DOI:** 10.3390/ijms19103227

**Published:** 2018-10-18

**Authors:** Agnieszka Walewska, Adam Szewczyk, Piotr Koprowski

**Affiliations:** Laboratory of Intracellular Ion Channels, Nencki Institute of Experimental Biology PAS, 3 Pasteur St., 02-093 Warsaw, Poland; a.walewska@nencki.gov.pl (A.W.); a.szewczyk@nencki.gov.pl (A.S.)

**Keywords:** mitochondria, potassium channels, KATP channel, BK_Ca_ channel, gasotransmitters, carbon monoxide, nitric oxide, hydrogen sulfide, heme

## Abstract

Recently, gaseous signaling molecules, such as carbon monoxide (CO), nitric oxide (NO), and hydrogen sulfide (H_2_S), which were previously considered to be highly toxic, have been of increasing interest due to their beneficial effects at low concentrations. These so-called gasotransmitters affect many cellular processes, such as apoptosis, proliferation, cytoprotection, oxygen sensing, ATP synthesis, and cellular respiration. It is thought that mitochondria, specifically their respiratory complexes, constitute an important target for these gases. On the other hand, increasing evidence of a cytoprotective role for mitochondrial potassium channels provides motivation for the analysis of the role of gasotransmitters in the regulation of channel function. A number of potassium channels have been shown to exhibit activity within the inner mitochondrial membrane, including ATP-sensitive potassium channels, Ca^2+^-activated potassium channels, voltage-gated Kv potassium channels, and TWIK-related acid-sensitive K^+^ channel 3 (TASK-3). The effects of these channels include the regulation of mitochondrial respiration and membrane potential. Additionally, they may modulate the synthesis of reactive oxygen species within mitochondria. The opening of mitochondrial potassium channels is believed to induce cytoprotection, while channel inhibition may facilitate cell death. The molecular mechanisms underlying the action of gasotransmitters are complex. In this review, we focus on the molecular mechanisms underlying the action of H_2_S, NO, and CO on potassium channels present within mitochondria.

## 1. Introduction

For a long period of time, gases such as carbon monoxide (CO), hydrogen sulfide (H_2_S), and nitric oxide (NO) were often considered to be toxic. However, much attention has only been paid to NO due to the fundamental role this gas plays in cardiovascular physiology. Although these gases often have pleiotropic targets, ion channels are probably one of the most important objects with which they interact. Hence, in this review, we describe the regulatory activities of gas molecules towards the class of potassium channels that are present in the inner mitochondrial membrane.

It is not feasible to directly record the activity of potassium channels in mitochondria due to the size of this organelle in comparison to the size of the recording pipette. Even the use of mitoplasts, spherical objects derived by the osmotic downshock of mitochondria stripped of its outer membrane leaving the inner membrane intact, in whole-mitoplast mode is challenging. Therefore, most of the data on mitochondrial ion channels were obtained from excised inner mitochondria membrane patches and require freshly isolated mitochondria. In addition, due to low success rate of such experiments in several studies model cell lines were used for which high expression of mitochondrial channels was established (as indicated in the text).

Potassium channels, including ATP-sensitive potassium channel (mitoK_ATP_), large-conductance calcium-activated potassium channel (mitoBK_Ca_) [1,2], voltage-gated potassium channels (mitoKv1.3 and mitoKv7.4), and TWIK-related acid-sensitive K^+^ channel 3 (mitoTASK-3), are present in the inner mitochondrial membrane [3]. They affect the integrity of mitochondrial inner membranes, leading to the regulation of energy-transducing processes and the synthesis of reactive oxygen species (ROS). Hence, these channels play an important role in cytoprotection [4,5,6] and constitute promising targets for cancer treatments [7,8,9]. It is important to mention that mitochondrial potassium channels are also present in plants [10] and simple organisms [11,12].

Mitochondrial potassium channels are modulated by inhibitors (channel blockers), such as glibenclamide and 5-hydroxydecanoic acid (5-HD), in the case of mitoK_ATP_ channels [13], or charybdotoxin, in the case of mitoBK_Ca_ channels [14]. Mitochondrial potassium channels are activated by potassium channel openers such as diazoxide (mitoK_ATP_ channels) [13] or NS1619 (mitoBK_Ca_ channels) [15]. In principle, all drugs acting on mitochondrial potassium channels have also been previously found to regulate plasma membrane potassium channels.

In this paper, we discuss the interactions of mitochondrial potassium channels with gasotransmitters such as nitric oxide (NO), carbon monoxide (CO), and hydrogen sulfide (H_2_S). We attempt to summarize the current gaps in our understanding of the nature of these interactions that may serve as future prospects for further study.

## 2. Carbon Monoxide

Carbon monoxide has been rediscovered in recent years as a vital mediator in the cell that regulates a wide variety of biochemical reactions. Fundamental to its function in both normal physiological processes and pathologies is its proficiency to regulate a number of ion channels, including members of the calcium-activated K^+^ (BK_Ca_) [16], voltage-activated K^+^ (Kv) [17] and Ca^2+^ channels [18], as well as ligand-gated P2X receptors [19]. The regulatory mechanisms by which CO modulates ion channels are still largely unexplained and stay somewhat dubious. However, the available structure-function studies suggest that a very limited range of protein cofactors confers indirect or direct sensitivity to CO, of a specific ion channel, and indicate that the redox state could play a crucial role in the integrated reaction. Irrespectively of the detailed mechanism by which ion channels are modulated by CO, its endogenous generation is physiologically important, and CO is currently being explored as a potential therapeutic [20].

### 2.1. Physiological Sources of Carbon Monoxide-Hemoxygenases

Historically, CO has been known to be toxic at high levels (>500 ppm). High CO concentrations cause hypoxemia via competitive binding, with an affinity that is >200-fold higher than that of oxygen, to hemoglobin in its binding sites for oxygen, with resulting formation of carboxyhemoglobin (CO-Hb) [21].

Endogenous CO is formed by oxidative decomposition of heme by heme oxygenases (HOs). In the presence of O_2_ and reduced nicotinamide adenine dinucleotide phosphate (NADPH), HOs catalyze the first, rate-limiting step in heme degradation, which is the breakdown of heme to equimolar amounts of biliverdin-IXα, iron, and CO [22].

Heme, which is a complex of iron and protoporphyrin IX, is an essential prosthetic group for a number of enzymes involved in the transport of oxygen (hemoglobin) and electrons (NADPH oxidase), as well as in other enzymes such as catalase and peroxidase [23]. However, heme functions not only as a prosthetic group within various proteins but has also been shown to function as a signaling molecule that regulates the functions of proteins, as described below.

Free heme released from intracellular heme-containing proteins causes cell damage and has been shown to have deleterious effects in several pathologies [24]. Heme levels have been found to be significantly increased in the cytosol and mitochondria of the failing heart [25]. The enzymatic reduction of biliverdin by biliverdin reductase produces bilirubin; both biliverdin and bilirubin are powerful antioxidants that scavenge ROS as part of a recycling mechanism [26]. Biliverdin reductase has also been found in mitochondria [27].

The role of free iron is complex. On one hand, at physiological levels, it plays a crucial role in cytoprotective processes [28], including ferritin-mediated processes that protect against mitochondrial dysfunction and oxidative stress [29]. On the other hand, there is evidence that iron produced from heme by HO may act as a pro-oxidant in lipid peroxidation and other reactions utilizing the Haber-Weiss cycle [30].

The high hydrophobicity of heme may promote deleterious iron-dependent reactions leading to the generation of reactive oxygen species (ROS) and membrane lipid peroxidation, thereby disrupting the cellular membranes of several organelles, including mitochondria [31]. Therefore, the levels of heme are tightly regulated via a balance of synthesis and degradation, and cells have developed heme detoxification systems [32]. In mammals, HOs are the only enzymes known to degrade heme, and therefore, they play a critical role in heme and iron homeostasis and are considered to be crucial components of the stress response and defenses against oxidative stress [33]. Three different isoforms of HO have been identified in mammals: inducible HO-1 [34]; constitutive HO-2 [35]; and catalytically non-active HO-3 [36]. HO-1 and HO-2 exhibit approximately 45% sequence identity.

HO-1 is induced by a variety of stimuli, including the presence of free heme, oxidative stress, and UV radiation [37,38,39]. HO-1 is localized to various intracellular compartments, including nuclei and mitochondria [27]. Mitochondria are hubs not only for heme but also for the synthesis of Fe-S cluster cofactors [40]; localization of the HO-1 protein to mitochondria suggests that it plays roles in regulation of turnover of heme proteins and iron in mitochondria, which may be important for protection against conditions, such as hemorrhage or ischemia/reperfusion, that have been found to involve the increased production of ROS. Consequently, increased expression of HO-1 may be observed in inflammatory and cardiovascular diseases [41,42].

A number of studies have demonstrated the beneficial role of HO-1 in mitochondria. Bindu et al. [43] showed that inducing oxidative stress by treating gastric mucosal cells with the non-steroidal anti-inflammatory drug indomethacin resulted in the up-regulation of HO-1 expression and the translocation of HO-1 to mitochondria. The presence of HO-1 in mitochondria has been linked to the inhibition of apoptosis during injury. In another study, it was shown that HO-1 was translocated to mitochondria and had increased activity in a primary culture of human small airway epithelial cells exposed to cigarette smoke extract, suggesting a protective effect of HO-1 against ROS [44].

However, not all the effects of HO-1 that were observed in mitochondria were beneficial. When HO-1 was specifically targeted to renal epithelial cell mitochondria [45], it mitigated hypoxia-mediated mitochondrial injury and cell death, but the selective advantages of its long-term expression were canceled out by its negative influence on the synthesis of mitochondrial proteins that contain heme as a cofactor. This conclusion was in line with the research of Bansal et al. [46], which showed that the overexpression of mitochondrial-targeted HO-1 resulted not only in heme degradation and diminished activity of heme-containing cytochrome c oxidase (CcO) but also induced the increased production of ROS and increased autophagy. Overall, these studies suggest that the level of mitochondrial-targeted HO-1 must be precisely controlled to achieve a balance between its beneficial and deleterious effects.

It has been suggested that HO-2 has different functions than HO-1 in the brain, based on differences in their patterns of expression. HO-1 has been shown to protect cultured cortical astrocytes, but not neurons, from oxidative stress after exposure to hemoglobin [47], whereas HO-2 has been shown to protect against apoptotic cell death in both neuronal cultures and in vivo models of ischemic injury [48]. HO-2 plays a unique role in oxygen sensing and responses to hypoxic conditions in mammalian cells [49]. There are three Cys-Pro signatures, called heme regulatory motifs (HRMs) in HO-2, which are not present in HO-1 [50]. The C-terminal HRM is a thiol/disulfide redox switch that modulates the affinity of the enzyme for heme [50] by responding to redox status of the cell, thereby integrating heme homeostasis with CO signaling and redox regulatory processes related to cellular metabolism. The oxygen sensor activity of HO-2 is involved in the regulation of the activity of the plasmalemmal BK_Ca_ channels within the carotid body, as described below. However, the possibility of localized functioning of HO-2 in mitochondria should also be noted; in one study, immunofluorescence staining of pulmonary arterial smooth muscle cells with various subcellular structural markers found that HO-2 was localized not only to the endoplasmic reticulum, but approximately 20% of the enzyme was located also in the mitochondria [51].

Although CO gas is easily diffusible and is able to reach all cellular compartments, the presence of HO-1 in mitochondria and its localized effects indicate that the interplay of CO with heme and possibly other products of HO activity, including biliverdin and free iron, is physiologically meaningful.

### 2.2. Signal Transduction via Heme-CO Interaction

Several bacterial nickel-dependent metalloenzymes are able to bind CO in their metal clusters, [52] but in mammalian cells, it is believed that the transduction of CO by proteins requires the presence of reduced heme iron (Fe^2+^). The first identified molecular target of CO was hemoglobin, but heme is also present in a number of electron transfer proteins and redox enzymes.

CO signaling pathways have still not been entirely elucidated. One of these pathways directly links the modulation of soluble guanylate cyclase (sGC) with the production of cyclic guanine monophosphate (cGMP) and the activation of protein kinase G (PKG) in processes involved in neurotransmission, vasodilation, and the inhibition of platelet aggregation [53,54,55,56]. CO-mediated activation of sGC is dependent on the presence of heme in its modulatory domain [57].

A few studies have provided evidence of the presence of sGC in the mitochondrial matrix. It was previously shown that the acceleration of cGMP production in cardiac mitochondria stimulates cytochrome c release in a manner that is independent of the permeability transition that results in apoptosis [58,59]. It has also been shown that protein kinase G-dependent opening of mitoBK_Ca_ channels plays a critical role in cardioprotection induced by sildenafil [60].

### 2.3. Regulation of BK_Ca_ Channels by CO

**Figure 1:**
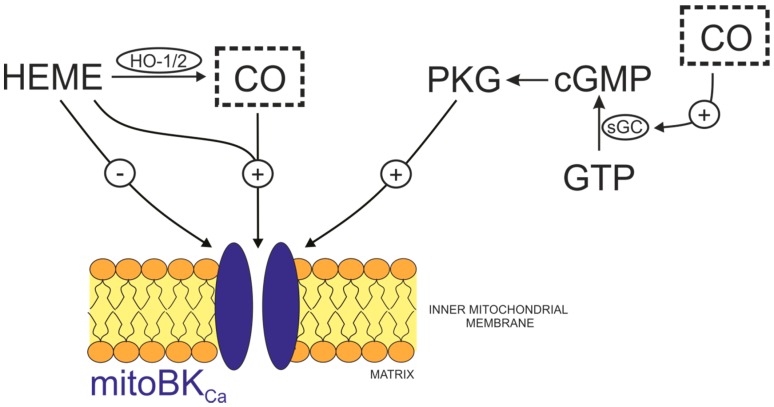
Regulation of the mitochondrial large-conductance calcium-activated potassium channel (mitoBK_Ca_) activity by CO. The activity of the channel is inhibited (−) by heme alone but could be stimulated (+) in the presence of heme (HEME, Fe-protoporphyrin IX) and CO (gasotransmitter shown in the dotted box) (a product of the activity of HOs). Independently, CO stimulates sGC/cGMP/PKG pathway leading to channel phosphorylation and activation. Abbreviations: CO, carbon monoxide; HO, heme oxygenase; PKG, cGMP-dependent protein kinase; sGC, soluble guanylate cyclase.

One of the best-characterized effectors of CO is the BK_Ca_ channel. It is known that CO activates this channel via direct and indirect mechanisms (Figure 1).

The indirect mechanism involves the phosphorylation of BK_Ca_ by PKG at specific serines (Ser855, Ser869, and Ser1072 in human BK_Ca_ ortolog Slo1), which increases the probability of BK_Ca_ assuming an open conformation [61,62,63]. Because CO stimulates sGC, leading to the activation of PKG, it is apparent that CO promotes PKG-mediated phosphorylation of BK_Ca_ channels and increases the channels’ activity. This mechanism appears to be valid also for mitoBK_Ca_ channels [64].

The direct mechanism involves the bimodal regulation of the BK_Ca_ channel via the interaction of CO with its bound heme [16]. Specifically, a segment that links regulators of K^+^ conductance (RCK) domains, RCK1 and RCK2, has been suspected to bind heme, based on the sequence homology of the short motif within this linker to the heme binding motif (HBM) containing the sequence C*XX*CH (*X* is any amino acid) that is present in cytochrome c [16]. Tang et al. [65] demonstrated that Fe^III^-heme (hemin) is able to block the BK_Ca_ channels with high affinity by binding to the HBM, since replacement of the Cys or His residues with Ser or Arg, respectively, abolished the sensitivity of the BK_Ca_ channel to hemin. Interestingly, cytochrome c covalently binds heme c via [66], while the noncovalent, reversible binding of heme b occurs within the BK_Ca_ channels [16]. The 23-residue peptide known as the heme-binding domain (HBD), which encompasses the HBM, preserves its heme- or hemin-binding properties [16,65]. It was demonstrated, using this model system, that heme, but not hemin, is able to bind CO [16]. Using a series of patch-clamp experiments, Jaggar et al. [16] demonstrated that BK_Ca_ channels that were inhibited by heme, but not hemin, could be reactivated by CO. The heme-CO complex exhibited stimulatory properties, since the channels were more active in the presence of heme-CO than in the control conditions. Thus, the substrate (heme) and the product (CO) of heme degradation by HOs regulated channel activity in such a way that an increase in the activity of HOs resulted in the activation of BK_Ca_ channels. In addition, using co-immunoprecipitation experiments, it was shown that HO-2 formed a complex with the BK_Ca_ channel by binding to the HBD, which provided evidence of a direct regulatory mechanism [67]. Within the CXXCH motif, the histidine residue serves as the axial heme ligand, while the cysteines comprise a thiol/disulfide redox switch that regulates the affinity of the HBM for heme and CO. The dithiol state was demonstrated to bind hemin (Kd = 210 nM) 14-fold more tightly than the disulfide state [68].

The thiol/disulfide redox switch within the BK_Ca_ channel and the corresponding thiol/disulfide redox switch within HO-2 are involved in hypoxic responses in the carotid body [69]. Carotid bodies are arterial chemoreceptors that sense changes in blood oxygen and CO levels and respond to hypoxia by secreting acetylcholine, dopamine, and ATP to increase the rate and depth of ventilation [69]. The hypoxia response element within the carotid body consists of glomus cells. BK_Ca_ channels on the plasma membranes of the glomus cells are inhibited when the O_2_ supply becomes compromised. Under these conditions, HO-2 activity is low due to the reduction of the cysteines within the HRM, which leads to lower levels of CO, higher levels of heme, and the binding of heme to the reduced form of the HBD in BK_Ca_ channels. This leads to BK_Ca_ inhibition, cell depolarization, Ca^2+^ influx, and transmitter release [67]. On the other hand, under normoxic conditions, CO generated by heme oxygenase-2 (HO-2) during heme degradation activates the BK_Ca_ channels, leading to cell hyperpolarization. This is a mechanism by which the BK_Ca_ channel responds in a prompt and reversible manner to alterations in the redox state of the cell, specifically as it shifts between hypoxia and normoxia [68].

An analogous mechanism, possibly resulting from the localization of both HO-2 and mitoBK_Ca_ inside mitochondria, could operate to control the redox state of mitochondria, which is of great importance. However, there is a lack of studies investigating this topic. In the only study, the effects of CORM-401 on glycolysis and respiration of mitochondria in intact human endothelial cell line EA.hy926 were studied [70]. It was found that CORM-401 treatment results in an increase in the oxygen consumption rate (OCR), inhibited glycolysis, decreased ATP-turnover, and increased proton leakage. Interestingly, the blockade of mitoBK_Ca_ with paxilline abolished the increase in OCR induced by CORM-401. Using patch-clamp experiments, the authors of this study showed that CORM-401 activated mitoBK_Ca_ channels. These effects were not observed for inactive CORM (iCORM) that was generated using a mix of MnSO_4_ and the CORM-401 ligand DTC. Therefore, it was argued that the observed effects specifically resulted from the CO released from the donor [70]. However, one must be cautious when interpreting experiments that involve CORMs due to a plethora of side effects that accompany the use of these drugs (see Section 2.5).

### 2.4. K_ATP_ Channels

Activation of mitoK_ATP_ has long been implicated in the protection of the heart against ischemia/reperfusion injury [13]. Abrogation of cardioprotection mediated by CORMs (CORM-2 and CORM-3) by the K_ATP_ channel inhibitors 5-hydroxydecanoic acid [71] or glibenclamide [72] provided indirect evidence that mitoK_ATP_ could be a target of CO. However, as was noted above, the action of CO requires the presence of heme as the receptor. Only recently was it discovered that cardiac plasmalemmal K_ATP_ channels are regulated by heme [73]. These channels are hetero-octameric complexes containing four pore-forming K^+^ channel subunits (Kir6.2) and four regulatory subunits (SUR2A) [74]. The modulation of the activity of this channel is important for the protective response of cardiac muscle to oxidative stress [75]. Curiously, it was found that a CXXHX_16_H motif in a cytoplasmic sulphonylurea receptor subunit within the channel is responsible for heme binding. In the presence of 500 nM hemin, a moderate (1.6-fold) increase in K_ATP_ receptor currents was observed [73]. A more dramatic, several-fold increase in the activation of K_ATP_ was observed in the presence of gaseous CO [76].

Unfortunately, there is no definitive evidence that identifies the proteins that constitute mitoK_ATP_.

A non-conventional short SUR2 splice variant approximately 55 kDa in size, known as SUR2A-55, was found predominantly within mitochondrial membrane fractions [77]. However, this variant does not contain the full-length SUR2A heme binding motif. Moreover, there is no consensus regarding the identity of the pore-forming subunit of mitoK_ATP_. Zhou et al. [78] used immunoelectron microscopy and found that Kir6.1 was mainly localized to the mitochondria, while Kir6.2 was located mainly within the endoplasmic reticulum with a minor fraction in the mitochondria. Similar results were observed upon the heterologous expression of Kir6.1-GFP [79]. On the other hand, other studies, including those utilizing cell fractionation, have excluded the presence of Kir6.1 or Kir6.2 in the mitochondria [80,81]. Recently, a splice variant of Kir1.1 lacking the first 19 N-terminal amino acids (designated Kir1.1b or ROMK2) was found to be located in mitochondria of H9c2 cell line and probably constitutes the pore-forming subunit of mitoK_ATP_ [82]. There are no data regarding the regulation of mitoK_ATP_ or Kir1.1 by gases, including CO.

### 2.5. Pharmacological CO Donors—CORMs

The experimental and clinical use of CO gas is difficult and can be dangerous. Therefore, the development of so-called CO-releasing molecules (CORMs) as prodrugs for the administration of CO in vitro, in tissue culture, and in living organisms (potentially as a medicine) resulted in a significant surge in the number of studies on this topic. CORMs offer the possibility of a safe and controllable release of CO at low amounts that is triggered by the presence of light, ligands, and enzymes, among other factors, with the ultimate goal of utilizing its therapeutic potential [83,84,85,86,87].

Despite the fact that a number of different CORM molecules are available, to date most studies have been conducted using various types of ruthenium-containing carbonyl complexes such as tricabonyldichlororuthenium(II) dimer (CORM-2) or [Ru(CO)_3_Cl(glycinate)] (CORM-3) [88]. Heme is generally considered to be the prime target of CO released from CORMs. However, it is not readily apparent whether or not any effect of a CORM is due to CO gas, the CORM itself, or one or more of its breakdown products. In one study that utilized heme-deficient bacteria, it was found that the response to CORM-3 could not be attributed to the classical biochemical targets of CO [89]. In another study, it was shown using crystallography that [Ru(CO)_3_Cl_2_(1,3-thiazole)] forms lysozyme-Ru adducts and that mono-carbonyl Ru species [Ru(H_2_O)_4_(CO)] were covalently bound to surface histidine and aspartates [90].

In several studies, it was shown that BK_Ca_ channels are strongly activated by CORM-2 or CORM-3 [91,92,93,94] and that different regions that are unrelated to the heme binding motif (HBM) were implicated in this phenomenon [95,96]. Specifically, the stimulatory action of CORM-2 on BK_Ca_ channels required the presence of an aspartic acid and two histidine residues (365 and 394) in the cytoplasmic RCK1 domain [96]. This effect persisted in conditions preventive for binding between CO and heme in other proteins. Only much later it was found that these histidines form Ru(CO) adducts, and the effects of CORM-2 were abolished in the presence of an excess of free histidine [97]. The authors of this study also uncovered the histidine-dependent action of CORM-2 on other voltage-gated potassium channels, such as Kv1.5 and Kv11.1, in a similar manner to that observed in Kv2.1 channels [98]. The off-site effects seen with CORM-2/CORM-3 were not observed for iron-containing CORM-S1 or manganese-containing CORM-EDE1 [97]. In contrast to the strong stimulatory effects of CORM-2, gaseous CO exerted only negligible effects on BK_Ca_ channels in a manner consistent with the targeting of heme by CO, which is only non-covalently and transiently bound to these channels [99] and occupies its binding sites probably only partially under experimental conditions.

The side effects of CORMs are unfortunately not restricted to ruthenium-based compounds. Manganese-containing CORM-401 stimulates respiration, depolarizes the cytoplasmic membrane in a manner similar to uncouplers, and elicits the loss of intracellular potassium in *E. coli* cells [100]. These properties could explain the increase of the oxygen consumption rate (OCR) in intact mitochondria [70] in the manner independent from the activation of mitoBK_Ca_. Therefore, it appears that there is a need to design and test other CO donors, including nonmetallic CORMs, e.g., CORM-A1 [101], to examine their impact on mitochondrial physiology. In addition, all of the effects reported to date should be verified with the use of gaseous CO [97].

## 3. Hydrogen Sulfide

Hydrogen sulfide (H_2_S), although considered to be a toxic gas for many years, was recently recognized to be an important molecule that, at low concentrations, plays an important role in processes such as inflammation, vasculature development [102,103], apoptosis [104], and the preservation of mitochondrial functioning [105,106].

### 3.1. Physiological Sources of Hydrogen Sulfide

Both enzymatic and non-enzymatic pathways contribute to the endogenous production of H_2_S. The following enzymes are involved in the generation of H_2_S from l-cysteine: cystathionine β-synthase (CBS), cystathionine γ-lyase (CSE), and 3-mercaptopyruvate sulfurtransferase (3-MST)/cysteine aminotransferase (CAT) [107]. In addition, it was shown that H_2_S can also be produced from d-cysteine by the 3-MST/DAO (d-amino acid oxidase) pathway in the kidney and the cerebellum [108]. CBS and CSE are distributed only in the cytoplasm [109], while CAT and 3-MST are found in both the cytoplasm and mitochondria, but primarily within mitochondria [110], and DAO is present in peroxisomes [111]. Although CSE is present only in the cytoplasm of vascular smooth muscle cells (SMCs) at rest, it can be translocated to the mitochondria [109] via the addition of the calcium ionophore A23187, thapsigargin, or tunicamycin to the SMCs. The increase in the amount of H_2_S in a mitochondrial fraction derived from A23187-treated SMCs was found to be CSE-related [109].

### 3.2. Pharmacological Donors of H_2_S

It is also possible to deliver H_2_S from exogenous sources, including H_2_S releasing molecules. The most popular of these are inorganic sulfide salts such as sodium sulfide (Na_2_S) and sodium hydrogen sulfide (NaHS) [112]. Although the positive effects of sulfide salts have been reported in relation to cardioprotection [113] and protection against inflammation [114,115], they release H_2_S rapidly and spontaneously, which impedes control of its concentration. Numerous other H_2_S-releasing molecules exist and each of them has both advantages and disadvantages, but they will not be addressed in this review [116]. Because we are focused here on the action of gas signaling molecules on mitochondrial potassium channels, we would like to briefly mention the novel mitochondrial-targeted H_2_S donors. AP39 and AP123 are mitochondrial slow-release H_2_S donors, which appear to be much safer than sulfide salts because they release concentrations that exert positive effects at a much lower level than that tolerated in vitro. AP39 and AP123 also have a beneficial effect on cellular bioenergetics by increasing the electron transfer rate of complex III of the respiratory chain [117].

### 3.3. H_2_S Regulation of Heme Proteins

The mechanisms underlying the action of H_2_S, which is complex and complicated, include redox reactions, the formation of persulfides with the -SH groups of cysteines and sulfide-metal interactions in heme proteins [118]. A few targets of H_2_S will be discussed below, with an emphasis on targets within mitochondria.

H_2_S reacts with the metallic centers of heme proteins. Coolman et al. [119], using a functional model of the oxygen-reducing sites within cytochrome c oxidase, showed that H_2_S acts as a competitive inhibitor of cytochrome c oxidase at high concentrations via its reversible binding to Fe^II^ heme, thus preventing binding of the enzyme substrate (O_2_) to the reduced Fe^II^Cu^I^ active site. At low concentrations, inhibitory effects of H_2_S on cytochrome c oxidase are not observed, due to the low affinity of H_2_S for Fe^II^ heme [119]. In addition to the inhibitory effects of H_2_S on cytochrome c oxidase, it was found that H_2_S (100 nM–1 µM) supports mitochondrial electron transport in cells isolated from rat liver [120]. H_2_S also increases OCR-linked respiratory capacity and ATP turnover in the presence of the Krebs-cycle-derived electron donor succinate. In addition, electron transport and production of ATP was stimulated by incubation of mitochondria from rat liver with low concentrations of 3-mercaptopyruvate (3-MP), which is a substrate for mitochondrial H_2_S production. Similar observations have been made based on mitochondria from cultured murine hepatoma cells [121]. H_2_S produced by 3-MST provides electrons to sulfide quinone oxidoreductase, which in turn transfers electrons to the mitochondrial electron transport chain and increases ATP production.

The above results indicate that H_2_S produced within the cell plays a physiological role in bioenergetics of the cell.

### 3.4. H_2_S Regulation via Protein S-Sulfhydration

Another mechanism by which H_2_S modifies the activity of proteins is S-sulfhydration (persulfidation) via the posttranslational modification of cysteine residues (RSH) into persulfides (RSSH) [122]. The ATP synthase (complex V) is one of the main proteins located within the inner membrane of mitochondria that undergoes S-sulfhydration [123]. This modification increases the enzymatic activity of ATP synthase in an NaHS concentration-dependent (10 nM–100 nM) manner. It was found that S-sulfhydration of the α subunit of ATP synthase (ATP5A1) occurs at Cys244 and Cys294. It is significant that the basal S-sulfhydration of ATP synthase is observed in vivo in wild-type mice and is induced by CSE-produced H_2_S, as this indicates the physiological importance of S-sulfhydration [123]. It is important to note that S-sulfhydration does not occur via the direct interaction of H_2_S and the -SH group of cysteine, because the sulfurs present in both molecules are typically at lowest i.e., (−2) oxidation state. However, there are other possible ways for the S-sulfhydration of cysteine residues to occur. For example, H_2_S can react with sulfenic acid (a product of -SH oxidation), S-nitrosated cysteines, and cysteine disulfides to form -SSH groups [124]. In addition, it has been shown that polysulfides can also take part in the S-sulfhydration of cysteine residues in vitro and in cell culture [125].

### 3.5. Potassium Channels as Targets for H_2_S

**Figure 2:**
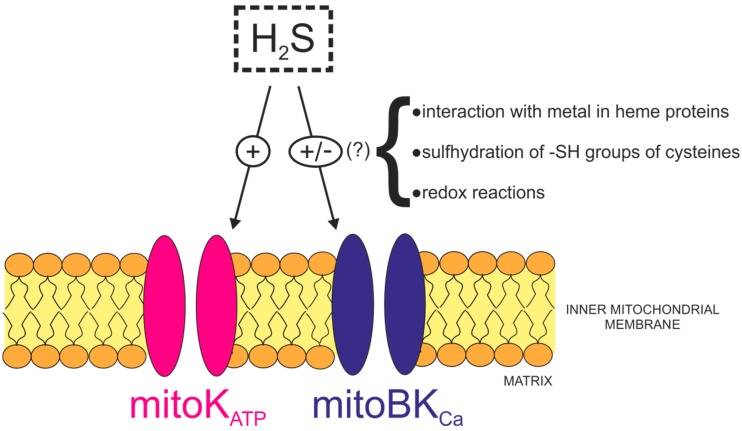
Regulation of mitochondrial potassium channels, mitochondrial large-conductance calcium-activated potassium channel (mitoBK_Ca_, activation (+) or inhibition (−)), and ATP-sensitive potassium channel (mitoK_ATP_, activation (+)) by H_2_S (gasotransmitter shown in the dotted box). Modulation of the activity of the mitoBK_Ca_ channel by H_2_S, inferred from regulation of its plasmalemmal counterpart, could result from multiple, unknown (?) mechanisms (bracket).

Hydrogen sulfide has various effects on the activity of different potassium channels within the plasma membrane (Figure 2) [126].

Mustafa et al. [127] showed in 2011 that the Cys43 in the Kir6.1 subunit of the K_ATP_ channel is a major target of H_2_S-mediated S-sulfhydration (NaHS was used as a donor) in HEK293 cells, which resulted in channel activation. Interestingly, an increase in the activity of ATP-sensitive potassium channels caused by S-sulfhydration was accompanied by reduced binding of ATP and enhanced binding of PIP_2_ to Kir6.1. It is possible that the S-sulfhydration of Cys43 in Kir6.1 impedes the binding of ATP to the channel due to electrostatic or spatial changes, which could result, in turn, in increased access of PIP_2_ to its binding site [127]. It was recently shown that H_2_S affects the activity of other Kir channels, Kir2 and Kir3, in a manner opposite to that of Kir6.1 mentioned above [128]. Specifically, the use of NaHS as an H_2_S donor resulted in the inhibition of Kir3.2 channels in *Xenopus* oocytes and Kir3.2 channels in CHO-K1 cells.

Tang et al. [129] revealed a direct effect of both exogenous H_2_S, in the form of an H_2_S-containing solution, and endogenous H_2_S on the activity of K_ATP_ channels in vascular smooth muscle cells. It was also shown that inward K_ATP_ currents and the probability of the opening of a single K_ATP_ channel was increased after exposure to H_2_S. To demonstrate the effects of endogenous H_2_S on the activity of K_ATP_ channels, various inhibitors of CSE and CBS were used, which resulted in a decrease of the whole-cell K_ATP_ currents in a manner independent on the activity of the cGMP signaling pathway [129].

In contrast, less is known about the regulation of the activity of potassium channels in the inner membrane of mitochondria by H_2_S. In 2016, Testai et al. [130] showed that 4-carboxyphenyl isothiocyanate (4CPI), which is an H_2_S donor, significantly improved the recovery of several parameters of myocardial function after ischemia and hindered the extent of tissue injury. Pre-treatment of rats with a selective blocker of the mitoK_ATP_ channel, 5-hydroxydecanoic acid (5-HD), lead to an abolition of the cytoprotective effects of 4CPI, which suggested that mitoK_ATP_ could be one of the targets of H_2_S donated by 4CPI. Additionally, the addition of 4CPI to isolated rat heart mitochondria caused depolarization of the mitochondrial membrane potential, and this effect was abrogated by ATP, which is a physiological blocker of the mitoK_ATP_ channel. A similar effect was observed in the action of NaHS on rat cardiomyocytes, where exposure to a H_2_S donor resulted in a reduction in the myocardial infarct size, which was in turn impaired by 5-HD [131]. From these experiments, it was inferred that H_2_S opened mitoK_ATP_ channels. Unfortunately, there are no electrophysiological data regarding the regulation of mitoK_ATP_ channels by H_2_S.

Similarly, nothing is known about the regulation of the activity of mitoBK_Ca_ channels by H_2_S. However, it is known that H_2_S regulates the activity of BK_Ca_ channels in the plasma membrane [132,133,134]. The effects of H_2_S on BK_Ca_ are equivocal and may vary in different tissues. The open probability (P_open_) and mean open time of a single BK_Ca_ channel in rat GH_3_ pituitary tumor cells was increased after NaHS exposure [132]. This effect was concentration- and voltage-dependent but also Ca^2+^-independent. In addition, an increase in the activity of the BK_Ca_ channel was found to be transient and reversible. Sitdikova et al. [132] also showed that the effects of NaHS on the activity of BK_Ca_ channels resulted from the reduction of sulfhydryl groups located on the cytoplasmic side of the channel by NaHS and found that H_2_S regulation is dependent on BK_Ca_ channel phosphorylation [133]. On the other hand, the activity of BK_Ca_ channels in HEK 293 cells was inhibited by NaHS in a dose-dependent manner [134].

It can be assumed that the mitoBK_Ca_ channels may be modulated by H_2_S in a similar manner to the BK_Ca_ channels within the plasma membrane, but the effects of H_2_S on these channels are still unknown. Because it is known that H_2_S interacts with heme proteins and that HBM is present in BK_Ca_ channels, a question arises as to whether or not H_2_S can impact the activity of these channels by interacting with heme bound to the HBM in a manner similar to the action of CO on BK_Ca_ channels [16].

## 4. Nitric Oxide

### 4.1. The Role and Physiological Sources of Nitric Oxide

Nitric oxide (NO) is a key signaling molecule in the cardiovascular system [135]. During ischemia, NO synthesis is increased in the heart to provide cardioprotection. NO is produced in vivo by nitric oxide synthase (NOS) via the conversion of l-arginine to citrulline [136]. NO plays a role in mitochondrial biogenesis and regulates mitochondrial turnover in the vasculature [137]. Interestingly, the alpha isoform of neuronal NOS-1 has been identified as a mitochondrial NOS (mtNOS) [138,139]. NO produced in mitochondria has an important regulatory impact on cellular metabolism via its reversible inhibition of cytochrome c oxidase [140]. Nitric oxide also reacts with mitochondrial superoxide anions to produce the potent oxidative species peroxynitrite [141], which hinders mitochondrial activities [142].

It is well known that activators of mitoK_ATP_ channels, such as diazoxide, can depolarize mitochondria [143]. However, it was found, unexpectedly, that diazoxide increased the phosphorylation of neuronal NOS at positive regulatory serine, 1417 but also decreased NOS phosphorylation at a negative regulatory Ser847, and, as a consequence, increased NO production in cultured neurons [144]. These observations functionally link mitochondrial channels with NO.

### 4.2. Regulation of Mitochondrial K_ATP_ Channels by Nitric Oxide

**Figure 3:**
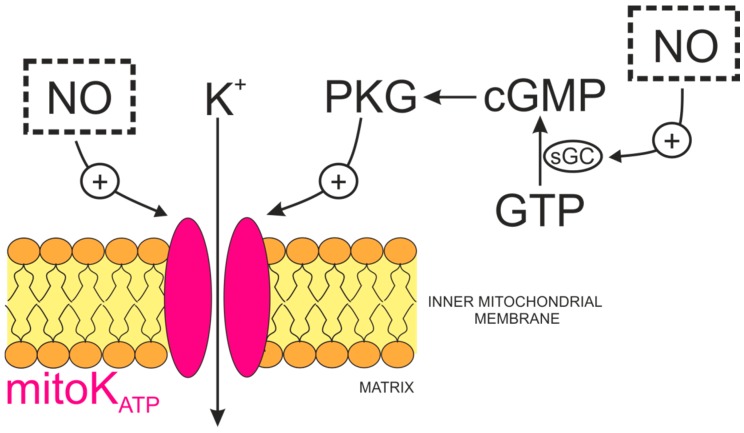
Regulation of the ATP-sensitive potassium channel (mitoK_ATP_) activity by NO. The activity of the channel is stimulated (+) directly by NO (gasotransmitter shown in the dotted box). Independently, NO could stimulate sGC/cGMP/PKG pathway leading to channel phosphorylation and activation. Abbreviations: PKG, cGMP-dependent protein kinase; sGC, soluble guanylate cyclase.

The cardioprotective properties of nitric oxide suggest that NO may interact with mitochondrial potassium channels (Figure 3).

By measuring mitochondrial redox potential and using this as an index of mitoK_ATP_ channel opening in rabbit ventricular myocytes, it was shown that NO may activate mitoK_ATP_ channels [145]. The NO donor S-nitroso-N-acetyl-DL-penicillamine (SNAP) activated mitoK_ATP_ without stimulating sarcolemmal K_ATP_ channels, since its effects were blocked specifically by 5-hydroxydecanoate (5-HD) and NO scavengers. Because many effects of NO are mediated by the cGMP-dependent pathway, it was determined whether or not NO-induced activation of mitoK_ATP_ was dependent on 8Br-cGMP. The negative results of this experiment suggested that mitoK_ATP_ are directly activated by NO. Interestingly, when activated by the potassium channel opener, diazoxide, mitoK_ATP_ channels appeared to be more susceptible to the potentiating effects of NO than channels that were closed [145]. Unambiguous evidence for the direct activation of mitoK_ATP_ by NO was obtained by reconstituting cardiac mitoK_ATP_ channels into lipid bilayers [146], whereupon the mitoK_ATP_ channels were activated by exogenous NO donors. Single channel activity was inhibited by the mitoK_ATP_ blockers 5-HD or glibenclamide [146]. In contrast, mitoK_ATP_ channel activity that was measured using the patch-clamp technique was inhibited by NO in human T-lymphocyte (Jurkat) cells [147]. Such differences could result from differences in the molecular mechanisms underlying the action of NO on mitoK_ATP_ channel proteins. Unfortunately, there are no mechanistic studies on mitochondrial channels that exist that could answer such questions. However, such differences could be elucidated via studies performed on plasmalemmal K_ATP_ channels. For example, cell-attached recordings of K_ATP_ currents in rat large DRG neurons showed that the K_ATP_ channels were stimulated by NO by decreasing their sensitivity to block by intracellular ATP [148]. This effect was also present after application of sGC and PKG inhibitors, indicating that the sGC/cGMP/PKG signaling pathway was not responsible for this phenomenon. This activation remained intact in inside-out patches and was reversed by dithiotreitol (DTT) and prohibited by a thiol-alkylating agent, NEM. These findings indicated that NO activates K_ATP_ channels via the direct S-nitrosylation of cysteine residues. During additional experiments in which the currents produced by recombinant SUR1/Kir6.2 channels expressed in COS7 cells were measured, proved that activation mediated by NO involves its interaction with residues in the NBD1 of the SUR1 subunit [148].

On the other hand, NO may also regulate mitoK_ATP_ channels indirectly via sGC/cGMP/PKG pathway [149]. For instance, in other studies that were performed in transfected HEK293 cells and in cardiomyocytes isolated from rabbits or genetically modified mice, exogenous exposure to the NO donor NOC-18 in cell-attached mode resulted in an increase of the single-channel activity of Kir6.2/SUR2A, which was abolished by exposure to a selective PKG inhibitor, KT5823 [150]. This sGC-dependent mechanism may likely be involved in the regulation of mitoBK_Ca_ channels in cardiomyocytes [64].

## 5. Final Remarks

In this paper, we have described our current understanding of the interactions of CO, H_2_S, and NO with mitochondrial potassium channels. Even so, our knowledge of these complex interactions is limited to phenomenology and is lacking molecular mechanisms. Identification of the molecular identity of mitochondrial potassium channels will increase insight into the interactions of gas molecules with mitochondrial channel proteins.

The interacting molecules of mitochondrial potassium channels are probably not limited to CO, H_2_S, and NO. The noble gas anesthetic, xenon, has been shown to protect the myocardium from ischemia/reperfusion injury [151], and this protection could possibly be mediated by the preservation of myocardial mitochondria and the opening of mitoK_ATP_ channels [152]. Additionally, sulfur dioxide (SO_2_) can also be generated endogenously in mammals and is a physiological endothelium-derived relaxing factor [153]. Moreover, the protective effect of SO_2_ on myocardial ischemia/reperfusion has been previously observed [154]. The vasodilatory effect of SO_2_ is related to the opening of K_ATP_ and BK_Ca_ channels in the plasma membrane [153]. This suggests that the regulation of mitochondrial potassium channels by SO_2_ is also possible. Other gaseous molecules, including ammonia (NH_3_), carbon dioxide (CO_2_), hydrogen gas (H_2_), and methane (CH_4_) have also been suggested to possibly serve as signaling molecules [155].

Increased understanding of the regulation of mitochondrial potassium channels by gas molecules will not only lead to increased knowledge of intracellular ion channels, but may also contribute to the future application of these substances and their donor molecules to cytoprotective treatment.
